# Perceived workload and heart rate variability in elite military pilots recovering from operational maintenance and air demonstration flights

**DOI:** 10.3389/fphys.2026.1919969

**Published:** 2026-09-23

**Authors:** Wilian de Jesus Santana, Gilberto Pivetta Pires, Diego Santiago, Edson Koury do Nascimento, Paulo Farinatti, Carlos Eduardo Rosa da Silva, Aylton Figueira Junior

**Affiliations:** 1 GETAFIS – Study Group on Training, Physical Activity, and Health, São Judas Tadeu University, São Paulo, Brazil; 2 Air Force University (UNIFA) – Brazilian Air Force University, Rio de Janeiro, Brazil; 3 LABSAU – Laboratory of Physical Activity and Health Promotion, Rio de Janeiro State University, Rio de Janeiro, Brazil

**Keywords:** aerobatic flight, heart rate variability, military aviation, NASA-TLX, occupational psychophysiology, perceived workload

## Abstract

**Introduction:**

Military pilots are exposed to high psychophysiological demands arising from Q9 environmental, operational, and cognitive factors. Heart rate variability (HRV) has been widely used as a non-invasive marker of autonomic modulation, whereas the NASA Task Load Index (NASA-TLX) allows the assessment of perceived workload. However, few studies have simultaneously investigated these parameters in aerial demonstration pilots.

**Objective:**

To compare autonomic responses assessed by HRV and perceived workload in pilots from the Brazilian Air Force Aerial Demonstration Squadron during recovery following operational maintenance (OM) and aerial demonstration (AD) flights.

**Methods:**

Nine elite military pilots (32 ± 3 years) performed two flights with an average duration of 50 minutes: an OM flight, characterized by lower operational complexity, and an AD flight, characterized by higher operational complexity and involving seven aircraft performing synchronized aerobatic maneuvers. HRV was recorded using the Equivital® system and analyzed with Kubios® software, including SDNN, RMSSD, LF, HF, and LF/HF during an immediate post-flight recovery segment. After each flight, perceived workload was assessed using the NASA-TLX. Comparisons between conditions were performed using the Wilcoxon signed-rank test, with statistical significance set at p < 0.05.

**Results:**

AD flights showed higher scores for temporal demand (+29%; p = 0.01) and effort (+28%; p = 0.03), as well as percentage increases in the other NASA-TLX dimensions. Most HRV indices remained stable between conditions (p > 0.05). The only significant difference was observed for maximum heart rate, which was 9% lower in the post-flight comparison between OM and AD conditions (p = 0.01).

**Discussion:**

Exploratory correlation analyses revealed sparse and largely non-significant associations between HRV indices and NASA-TLX dimensions, with only a few nominal associations involving temporal demand and the spectral HF component.

**Conclusion:**

Aerial demonstration flights were associated with higher perceived workload scores, particularly in the temporal demand and effort dimensions. However, no statistically significant differences were detected for most HRV indices assessed during the immediate post-flight recovery period. These findings suggest relative stability of autonomic responses in highly experienced military pilots, even in the presence of increased perceived workload.

## 1 Introduction

Military flight operations expose pilots to high operational demands resulting from the combined physical, cognitive, and environmental stressors present during flight. In high-performance aircraft, particularly during aerobatic maneuvers and operations involving high gravitational loads, aviators are subjected to substantial physiological and autonomic challenges that may influence their ability to adapt to the operational environment (Convertino, 1999; Petrassi et al., 2012; Hargens et al., 2013; Choi et al., 2015).

Heart rate variability (HRV) has been widely used as a non-invasive marker of cardiovascular autonomic modulation and adaptive capacity in response to different operational stressors. In aviation settings, changes in HRV indices have been associated with mental workload, task complexity, operational stress, and in-flight performance (Alaimo et al., 2020; Wang et al., 2024; Soares et al., 2024). However, the literature has reported heterogeneous findings, suggesting that increases in perceived workload are not always accompanied by proportional changes in traditional autonomic markers, particularly among highly trained and experienced pilots (Wang et al., 2024; Koskelo et al., 2024).

In parallel, perceived workload represents an important component of human factors related to performance and operational safety. Among the available instruments, the NASA Task Load Index (NASA-TLX) is widely used to quantify different workload dimensions, including mental demand, physical demand, temporal demand, effort, performance, and frustration (Hart and Staveland, 1988). Recent evidence indicates that integrating physiological and perceptual measures provides a more comprehensive understanding of human responses in complex operational environments than the isolated use of either type of indicator (Alaimo et al., 2020; Mansikka et al., 2019; Masi et al., 2023).

Although the monitoring of workload and autonomic modulation has received increasing attention in the aviation literature, most studies have been conducted in flight simulators or during conventional operational missions (Mohanavelu et al., 2020; Koskelo et al., 2024; Wang et al., 2024). Consequently, evidence obtained under real flight conditions involving aerial demonstration pilots remains scarce. This is a highly specialized population exposed to unique operational demands arising from the coordinated execution of formation aerobatic maneuvers under strict temporal constraints.

The Brazilian Air Force Aerial Demonstration Squadron represents a particularly relevant context for investigating these responses, as its pilots perform both operational maintenance flights, characterized by lower complexity and individual execution, and aerial demonstration flights, which involve multiple aircraft, high flight precision, and intense interpersonal coordination. Comparing these conditions provides a unique opportunity to understand how different levels of operational complexity simultaneously influence perceived workload and autonomic modulation in a real flight environment.

Although the present findings are specific to the Brazilian Air Force Aerial Demonstration Squadron, the broader pattern observed here — elevated perceived workload alongside relatively stable post-recovery autonomic indices in highly trained personnel — may offer conceptual insight for HRV-based workload monitoring, fatigue risk management, and training protocols in other elite or high-demand operational settings, such as civil aviation, motorsport, and high-performance occupational contexts, where similar dissociations between subjective and autonomic indicators could plausibly occur.

Considering the operational differences between Operational Maintenance (OM) and Air Demonstration (DA) flights, this study was developed to answer three main questions: (i) does the perceived workload differ between the two flight profiles?; (ii) does cardiac autonomic recovery immediately after landing differ between these operating conditions?; and (iii) are there associations between the dimensions of perceived workload and heart rate variability indices during the post-flight recovery period? Based on the greater operational complexity of air demonstration flights, it was hypothesized that this condition would be associated with higher perceived workload scores and differences in the autonomic indicators assessed during post-flight recovery. Thus, the objective of this study was to compare heart rate variability and perceived workload during recovery after operational maintenance and aerial demonstration flights performed by pilots of the Brazilian Air Force Air Demonstration Squadron.

## 2 Materials and methods

### 2.1 Study design

This observational study adopted a repeated-measures, within-subject design conducted in a real operational environment. Each pilot was assessed under two conditions: operational maintenance, characterized by lower operational complexity, and aerial demonstration, characterized by greater complexity, multi-aircraft formation, and synchronized maneuvers.

### 2.2 Participants

The sample consisted of nine male pilots (32.1 ± 1.8 years), all active members of the Brazilian Air Force Aerial Demonstration Squadron. The exclusively male composition of the sample reflected the characteristics of the operational personnel available during the data collection period. Pilots were included if they were engaged in regular operational activity within the squadron and were clinically cleared for flight. Individuals using central nervous system stimulants or presenting temporary medical restrictions were excluded.

Despite the small sample size, the participants represented approximately 60% of the squadron’s operational personnel, which consisted of 15 pilots. This characteristic confers high ecological relevance to the findings, as they were obtained in an operational context that is difficult to access for scientific investigation.

### 2.3 Procedures

All procedures were performed using the A-29 Super Tucano aircraft (EMB-314), which is used for advanced training and tactical missions. Data collection occurred in three stages: anthropometric assessment and estimation of maximal oxygen uptake using the Yo-Yo Intermittent Recovery Test; execution of the operational maintenance (OM) flight, characterized by lower technical complexity and maneuvers performed individually; and execution of the aerial demonstration (AD) flight, involving a seven-aircraft formation, high synchronization demands, and greater psychophysiological load (**Figure 1**). Operational maintenance flights are carried out individually, focusing on maintaining the operational proficiency of the aircraft and the pilot, involving less complexity of interpersonal coordination and execution of less demanding maneuvers from the point of view of operational synchronization. In contrast, air demonstration flights are conducted in formation with seven aircraft simultaneously, requiring strict time synchronization, continuous monitoring of the relative position of the remaining aircraft, and precise execution of collective aerobatic maneuvers. Although objective measurements of gravitational acceleration were not recorded during the missions, these operational characteristics justify the expectation of greater workload perceived during the aerial demonstration flights.

**Figure 1:**
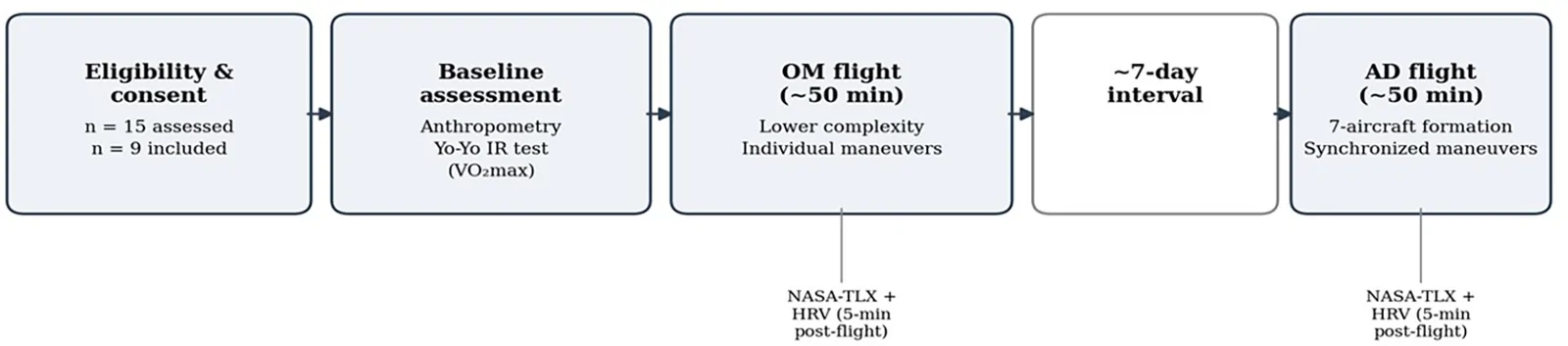
Overview of the study procedure, including eligibility screening, baseline assessment, and the sequence of operational maintenance (OM) and aerial demonstration (AD) flights, each followed by NASA-TLX administration and a 5-minute post-flight HRV recording.

All sessions followed the squadron’s standardized operational protocols. However, because the study was conducted in a real operational environment, variables such as time of day, sleep, food intake, hydration status, and previous operational load could not be fully controlled. During the second session, the OM flight lasted approximately 50 minutes, during which pilots maintained their usual routines, including the intake of food, beverages, and prescribed medications. Maneuvers were performed according to the squadron’s operational guidelines.

During the AD mission, seven aircraft flew in synchronized formation while performing complex group and individual maneuvers. The OM flight was considered the reference condition, whereas the AD flight represented the experimental condition. Workload was assessed immediately after each flight using the NASA-TLX instrument. HRV was recorded using the Equivital^®^ system. Each pilot wore the device approximately 15 minutes before flight and continued wearing it for 30 minutes after landing. Both flight conditions had an average duration of 50 minutes and were conducted at different times of the day, with a one-week interval between them. Although electrocardiographic recordings were obtained continuously throughout the flight period, the present investigation focused specifically on the analysis of the five minutes immediately after landing. This methodological decision was adopted to allow direct comparison between the heart rate variability indices and the workload scores obtained using the NASA-TLX, an instrument applied immediately after each mission. Thus, the physiological and perceptual indicators refer to the same temporal context of post-flight recovery. In addition, the use of five-minute stationary segments is in line with international recommendations for short-term analyses of heart rate variability (Task Force of the European Society of Cardiology and the North American Society of Pacing and Electrophysiology, 1996; Laborde et al., 2017).

### 2.4 Instruments

#### 2.4.1 Psychometric instrument

The NASA-TLX is an instrument composed of six subscales or dimensions: mental demand, physical demand, temporal demand, performance, effort, and frustration (Diniz, 2003; Cardoso and Gontijo, 2012). Developed by Hart and Staveland (1988), the NASA-TLX has been widely used to estimate workload in flight simulators (Mansikka et al., 2019; Rainieri et al., 2021), commercial aviation flights (Wanyan et al., 2014), and military flight operations (Orlandi and Brooks, 2018). The method is based on self-assessment, in which participants subjectively rate their exposure to workload across the six aforementioned dimensions (Casner and Gore, 2010). In the present study, the unweighted version of the NASA-TLX was used, comprising six dimensions rated on a 0- to 20-point scale. The score for each dimension was analyzed separately, and the global score, when applicable, was calculated as the mean of the six dimensions. The unweighted version was adopted because it substantially reduces respondent burden relative to the original weighted procedure, while showing comparable sensitivity and psychometric performance in most applications, including operational and aviation settings (Hart, 2006).

#### 2.4.2 Heart rate variability assessment

HRV was assessed using the Equivital^®^ system (Hidalgo Ltd., Cambridge, United Kingdom), consisting of a physiological vest capable of continuously recording electrocardiographic signals, heart rate, respiratory rate, skin temperature, triaxial acceleration, and variables related to body position. The system has internal memory for data storage throughout the activity, allowing continuous acquisition even in environments without direct connection to a computer. Previous studies have demonstrated its validity and applicability for physiological monitoring in different occupational and sports contexts (Liu et al., 2013; Ferguson and Myers, 2018; Ferguson et al., 2019).

The electrocardiographic signal was acquired at a sampling frequency of 256 Hz. RR intervals were obtained from the electrocardiographic signal recorded by the Equivital^®^ system and subsequently exported for HRV analysis using Kubios HRV Scientific Lite software, version 4.1.0 (University of Eastern Finland, Kuopio, Finland).

For each flight, a five-minute segment immediately after aircraft landing was selected, in accordance with international recommendations for short-term HRV analysis (Task Force of the European Society of Cardiology and the North American Society of Pacing and Electrophysiology, 1996; Laborde et al., 2017). The recordings showed high signal quality, and no manual correction of RR intervals was required. Automatic artifact correction was performed using the threshold-based correction filter set to Very Low, resulting in 0% corrected beats. Detrending was performed using the smoothness priors method, with λ = 500, and the data were interpolated at a frequency of 4 Hz.

Spectral analysis was performed using the Fast Fourier Transform (FFT), with 300-second windows and 50% overlap. The spectral bands adopted were very-low-frequency power (VLF: 0.00–0.04 Hz), low-frequency power (LF: 0.04–0.15 Hz), and high-frequency power (HF: 0.15–0.40 Hz), according to international standards for HRV studies (Task Force of the European Society of Cardiology and the North American Society of Pacing and Electrophysiology, 1996). LF and HF spectral components were expressed in absolute units (ms²), and the LF/HF ratio was also calculated.

HRV indices were analyzed in the time, frequency, and nonlinear domains. In the time domain, the standard deviation of normal RR intervals (SDNN) and the root mean square of successive differences between adjacent RR intervals (RMSSD) were obtained. In the frequency domain, LF, HF, and the LF/HF ratio were analyzed. Nonlinear analysis included indices derived from the Poincaré plot, particularly the SD2/SD1 ratio, which was used as a complementary indicator of cardiac autonomic dynamics. Additionally, minimum heart rate (HRmin), mean heart rate (HRmean), and maximum heart rate (HRmax) were recorded during each flight. It should be noted that SDNN reflects overall RR interval variability, including contributions from very-low-frequency oscillations that require longer recording periods (ideally 24-hour recordings) to be fully captured and meaningfully interpreted (Task Force of the European Society of Cardiology and the North American Society of Pacing and Electrophysiology, 1996). Although SDNN was retained in the present analysis to allow comparability with previous short-term HRV studies conducted in military and aeronautical populations, its interpretive validity in five-minute segments is comparatively limited relative to RMSSD, which is considered more robust for short-term vagal assessment.

### 2.5 Statistical analysis

Statistical power analysis was performed using G*Power software, version 3.1.9.4, considering a paired-samples t-test, moderate effect size, 5% significance level, 80% statistical power, and a one-tailed hypothesis. The sample size calculation indicated that 27 participants would be required to achieve 80% statistical power. However, due to operational constraints inherent to the population investigated, only nine pilots could be assessed. Therefore, the results should be interpreted with caution, considering the potential limitation in detecting small or moderate differences between the investigated conditions. As data normality was rejected, a nonparametric approach was adopted, using the Wilcoxon signed-rank test for paired comparisons.

Descriptive statistics and percentage changes between the OM and AD conditions were calculated. Effect size was determined by dividing the Z value by the square root of the sample size, with interpretation based on Cohen (1988) Statistical analyses were performed using SPSS 25.0, and graphical representations were generated using GraphPad Prism 8.0.2. Statistical significance was set at p < 0.05. Associations between HRV indices and NASA-TLX dimensions were assessed using Spearman’s rank correlation coefficient, given the non-normal distribution of the data and the small sample size. Correlation magnitudes were interpreted according to Cohen’s (1988) criteria; however, given the reduced statistical power inherent to n = 9 (df = 7), these correlations should be regarded as exploratory and hypothesis-generating rather than confirmatory. No correction for multiple comparisons (e.g., Bonferroni or false discovery rate) was applied to the pairwise comparisons or correlation analyses. This decision was based on the exploratory nature of the study and the already limited statistical power resulting from the small sample size; applying conservative corrections would further increase the risk of Type II errors, potentially obscuring meaningful associations. Consequently, all p-values presented should be interpreted as nominal and exploratory rather than confirmatory, and findings require replication in adequately powered samples.

### 2.6 Ethics statement

The study involving human participants was approved in accordance with Brazilian National Health Council Resolution No. 466/12 and complied with the ethical principles established by the Ethics Committee of São Judas University, under approval number 5.786.099. All procedures were conducted in accordance with current legislation and applicable institutional requirements. All participants provided written informed consent to participate in the study. In addition, written consent was obtained from the individuals for the publication of any potentially identifiable images or data included in this article.

## 3 Results

Initially, 15 participants were assessed for eligibility. Of these, six were excluded because they did not meet the inclusion criteria, either due to lack of clinical clearance for flight activity or because they were unavailable as a result of operational assignments at other bases. Thus, the final sample consisted of nine pilots. The recruitment process and the experimental protocol were conducted over a 24-month period, with all data collection completed in November 2023.

The descriptive characteristics of the sample are presented in **Table 1**. The pilots showed an anthropometric profile compatible with the operational requirements of their role and had extensive flight experience. Flight experience was controlled according to the aircraft in which data collection was performed, although some participants had additional flight experience in other types of aircraft.

**Table 1:** Demographic characteristics of the Brazilian Air Force pilot sample.

Characteristic	Value
Sample size	9 pilots
Age (years)	32 ± 3
Body mass (kg)	73.7 ± 8.1
Height (cm)	178 ± 7
Body fat (%)	17.4 ± 4.0
VO_2_max (mL·kg^-^¹·min^-^¹)	41.6 ± 3.0
Flight experience (hours)	1,640 ± 1,058

Values are presented as mean ± standard deviation, except for sample size.

The comparison of perceived workload between operational maintenance (OM) and aerial demonstration (AD) flights is presented in **Table 2**. Overall, AD flights showed higher scores in almost all NASA-TLX dimensions, indicating an increase in the operational demands perceived by the pilots. Statistically significant differences were observed for temporal demand and effort, whereas the remaining dimensions showed an upward trend without reaching statistical significance.

**Table 2:** Comparison of NASA-TLX scores after operational maintenance and aerial demonstration flights.

Dimension	OM mean ± SD	AD mean ± SD	OM min–max	AD min–max	OM median [P25–P75]	AD median [P25–P75]	% change OM–AD	Z	p-value	r
Mental demand	11.4 ± 3.6	14.2 ± 3.1	5.0–16.0	10.0–19.0	12.0 [8.5–14.5]	13.0 [12.0–17.0]	+24%	-1.72	0.09	0.57
Physical demand	10.1 ± 3.2	13.3 ± 3.5	5.0–15.0	9.0–18.0	11.0 [7.5–12.5]	12.0 [10.0–16.5]	+32%	-1.72	0.09	0.57
Temporal demand	8.8 ± 3.2	11.3 ± 1.9	2.0–12.0	8.0–13.0	9.0 [7.0–11.5]	12.0 [9.5–13.0]	+29%	-2.54	0.01*	0.85
Performance	12.8 ± 3.3	15.2 ± 2.5	7.0–18.0	10.0–18.0	14.0 [10.5–15.0]	15.0 [14.0–17.5]	+19%	-1.85	0.06	0.62
Effort	10.9 ± 2.6	13.9 ± 2.6	5.0–14.0	11.0–19.0	11.0 [10.0–12.5]	14.0 [11.5–15.5]	+28%	-2.20	0.03*	0.73
Frustration	7.4 ± 4.7	4.9 ± 4.0	1.0–14.0	1.0–13.0	6.0 [4.0–13.0]	3.0 [2.5–8.0]	-34%	-1.52	0.13	0.51

Data are presented as mean ± standard deviation, minimum and maximum values, and median [25th–75th percentiles]. OM, operational maintenance; AD, aerial demonstration; NASA-TLX, NASA Task Load Index; SD, standard deviation. Comparisons between OM and AD conditions were performed using the Wilcoxon signed-rank test. Percentage change refers to the relative change from OM to AD. r, effect size, calculated as |Z|/√N (Cohen, 1988). *p < 0.05.

Mental demand increased by 24% in the aerial demonstration condition, from 11.4 ± 3.6 during operational maintenance to 14.2 ± 3.1 during aerial demonstration, although this difference did not reach statistical significance (Z = -1.72; p = 0.09). A similar pattern was observed for physical demand, which increased by 32%, from 10.1 ± 3.2 to 13.3 ± 3.5 (Z = -1.72; p = 0.09).

Temporal demand differed significantly between the assessed conditions, increasing by 29% during aerial demonstration flights. Mean scores increased from 8.8 ± 3.2 during operational maintenance to 11.3 ± 1.9 during aerial demonstration (Z = -2.54; p = 0.01). The performance dimension also increased by 19%, with mean scores of 12.8 ± 3.3 in the OM condition and 15.2 ± 2.5 in the AD condition, although this difference was not statistically significant (Z = -1.85; p = 0.06).

The effort dimension showed a significant increase of 28% after aerial demonstration flights, from 10.9 ± 2.6 to 13.9 ± 2.6 (Z = -2.20; p = 0.03). In contrast, frustration decreased by 34% during aerial demonstration, from 7.4 ± 4.7 in the operational maintenance condition to 4.9 ± 4.0 in the aerial demonstration condition, with no statistically significant difference (Z = -1.52; p = 0.13).

Overall, these results indicate that aerial demonstration flights were accompanied by increased cognitive, physical, and temporal demands perceived by the pilots, particularly reflected by significant increases in temporal demand and effort.

In addition to the analysis of each NASA-TLX dimension separately, a global workload score was calculated as the unweighted mean of the six dimensions for each pilot and condition. The global score showed a 17.2% increase from the OM to the AD condition (10.46 ± 2.69 vs. 12.26 ± 2.01), which approached but did not reach conventional statistical significance (Z = -1.96; p = 0.050; r = 0.65). Although this result did not cross the p < 0.05 threshold, the large effect size is consistent with the pattern observed for the individual dimensions that reached significance (temporal demand and effort), suggesting a trend toward higher overall perceived workload during aerial demonstration flights. This borderline result may reflect, in part, the limited statistical power associated with the sample size (n = 9), although this explanation was not directly tested (e.g., via *post-hoc* power analysis) and should be regarded as a plausible interpretation rather than a demonstrated finding.

Post-flight HRV data for the operational maintenance (OM) and aerial demonstration (AD) conditions are presented in **Table 3**. Overall, autonomic indices remained relatively stable across the different operational contexts, as most of the variables analyzed did not differ significantly between conditions.

**Table 3:** Comparison of post-flight heart rate variability indices between operational maintenance and aerial demonstration flights.

Variable	OM mean ± SD	AD mean ± SD	OM min–max	AD min–max	OM median [P25–P75]	AD median [P25–P75]	% change OM–AD	Z	p-value	r
Mean RR interval (ms)	678 ± 84.1	690 ± 93.2	540–867	481–895	662 [615–759]	690 [622–759]	+2%	-0.94	0.34	0.31
SDNN (ms)	43.63 ± 20.23	42.96 ± 11.24	10.45–92.63	21.57–67.92	42.56 [31.82–52.04]	42.33 [34.68–51.12]	-2%	-0.14	0.89	0.05
HRmean (bpm)	90 ± 11	88 ± 13	69–112	67–125	91 [79–98]	87 [79–97]	-2%	-1.18	0.26	0.39
HRmin (bpm)	71 ± 9	71 ± 8	52–86	61–93	73 [63–76]	70 [65–75]	0%	-0.266	0.79	0.09
HRmax (bpm)	120 ± 26	109 ± 19	88–190	77–183	113 [104–121]	106 [96–117]	-9%	-2.410	0.01*	0.80
RMSSD (ms)	32.16 ± 17.41	31.10 ± 10.17	12.04–89.42	14.57–48.80	27.42 [20.81–39.20]	30.77 [21.47–39.62]	-3%	-0.457	0.64	0.15
LF (ms²)	0.091 ± 0.028	0.088 ± 0.032	0.040–0.150	0.040–0.211	0.090 [0.070–0.115]	0.087 [0.067–0.100]	-3%	-0.737	0.46	0.25
HF (ms²)	0.183 ± 0.044	0.183 ± 0.065	0.150–0.337	0.150–0.482	0.163 [0.157–0.189]	0.160 [0.150–0.183]	0%	-0.457	0.64	0.15
LF/HF	0.435 ± 0.235	0.523 ± 0.327	0.116–1.124	0.162–1.362	0.345 [0.272–0.581]	0.437 [0.291–0.580]	+20%	-0.976	0.32	0.33
SD2/SD1	2.604 ± 0.793	2.658 ± 0.466	1.100–3.900	1.900–3.700	2.700 [2.000–3.150]	2.600 [2.300–2.950]	+2%	-0.131	0.89	0.04

Data are presented as mean ± standard deviation, minimum and maximum values, and median [25th–75th percentiles]. OM, operational maintenance; AD, aerial demonstration; HRV, heart rate variability; SD, standard deviation; RR, interval between consecutive R waves; SDNN, standard deviation of normal RR intervals; HRmean, mean heart rate; HRmin, minimum heart rate; HRmax, maximum heart rate; RMSSD, root mean square of successive differences between adjacent RR intervals; LF, low-frequency power; HF, high-frequency power; LF/HF, ratio between low- and high-frequency components; SD2/SD1, ratio between the longitudinal and transverse axes of the Poincaré plot. Comparisons between OM and AD conditions were performed using the Wilcoxon signed-rank test. Percentage change refers to the relative change from OM to AD. r, effect size, calculated as |Z|/√N (Cohen, 1988). *p < 0.05.

Mean RR intervals showed a slight increase of 2% after aerial demonstration flights, from 678 ± 84.1 ms during operational maintenance to 690 ± 93.2 ms during aerial demonstration (Z = -0.94; p = 0.34). In contrast, global HRV indices showed small reductions. SDNN changed from 43.63 ± 20.23 ms to 42.96 ± 11.24 ms, corresponding to a 2% decrease (Z = -0.14; p = 0.89), whereas RMSSD decreased by 3%, from 32.16 ± 17.41 ms in the OM condition to 31.10 ± 10.17 ms in the AD condition (Z = -0.457; p = 0.64).

A similar pattern was observed for mean heart rate, which decreased slightly from 90 ± 11 bpm to 88 ± 13 bpm between the assessed conditions (Z = -1.18; p = 0.26). Minimum heart rate, on the other hand, remained virtually unchanged, with a mean value of 71 bpm in both operational maintenance and aerial demonstration conditions (Z = -0.266; p = 0.79).

Among all variables investigated, only HRmax differed significantly between flight types. Mean values decreased from 120 ± 26 bpm during operational maintenance to 109 ± 19 bpm during aerial demonstration, corresponding to a 9% reduction (Z = -2.410; p = 0.01).

In the spectral analysis, LF and HF components remained relatively consistent across operational conditions. LF changed from 0.091 ± 0.028 in the OM condition to 0.088 ± 0.032 in the AD condition, representing a 3% reduction (Z = -0.737; p = 0.46), whereas HF remained at a mean value of 0.183 in both conditions (Z = -0.457; p = 0.64). Although not statistically significant, the LF/HF ratio increased by approximately 20% during aerial demonstration flights, from 0.435 ± 0.235 to 0.523 ± 0.327 (Z = -0.976; p = 0.32). The SD2/SD1 ratio showed only a small increase between the assessed contexts, from 2.604 ± 0.793 to 2.658 ± 0.466 (Z = -0.131; p = 0.89).

Taken together, these results did not show statistically significant differences for most autonomic indices assessed between the operational conditions investigated.

To further characterize the temporal dynamics of autonomic responses beyond the immediate post-flight recovery period, a complementary exploratory analysis was conducted using continuously recorded RR intervals segmented into three physiological phases: rest (pre-flight baseline), reactivity (mean of three 5-minute in-flight windows), and recovery (post-flight, as reported in **Table 3**). Because this analysis used unprocessed RR intervals rather than the Kubios-corrected indices reported in **Table 3**, absolute values differ slightly from those previously described; however, within-phase comparisons between OM and AD conditions remain valid, as any processing-related bias would apply equally to both conditions. No HRV index differed significantly between OM and AD during the rest phase (all p > 0.21) or the reactivity phase (all p > 0.42). During recovery, HRmax showed the same directional pattern reported in **Table 3** (lower in AD), approaching significance (Z = -1.84; p = 0.066; r = 0.61). Taken together, these findings indicate that cardiac autonomic responses remained statistically comparable between operational conditions not only during post-flight recovery, but also at rest and during the in-flight reactivity phase, reinforcing the interpretation of relative autonomic stability across the entire psychophysiological trajectory of the mission, despite the increase in perceived workload.

The correlations between HRV indices and NASA-TLX workload dimensions after operational maintenance (OM) flights are presented in **Table 4**. Overall, low to moderate associations were observed between autonomic parameters and NASA-TLX components.

**Table 4:** Correlations between HRV indices and NASA-TLX dimensions after operational maintenance flights.

Variable	Mental demand	Physical demand	Temporal demand	Performance	Effort	Frustration
SDNN	-0.050 (p=0.898)	-0.102 (p=0.794)	-0.135 (p=0.729)	-0.135 (p=0.729)	-0.409 (p=0.274)	-0.244 (p=0.527)
HRmean	-0.217 (p=0.575)	0.203 (p=0.600)	-0.219 (p=0.571)	-0.219 (p=0.571)	0.085 (p=0.828)	0.034 (p=0.931)
HRmin	-0.100 (p=0.798)	-0.170 (p=0.662)	-0.667 (p=0.050)*	-0.169 (p=0.664)	-0.204 (p=0.599)	-0.454 (p=0.220)
HRmax	0.083 (p=0.832)	0.119 (p=0.760)	0.203 (p=0.600)	0.101 (p=0.796)	0.426 (p=0.253)	0.328 (p=0.389)
RMSSD	0.133 (p=0.733)	0.203 (p=0.600)	0.650 (p=0.058)	-0.135 (p=0.729)	0.272 (p=0.479)	0.639 (p=0.064)
LF	-0.400 (p=0.286)	0.119 (p=0.760)	-0.186 (p=0.632)	0.397 (p=0.290)	0.043 (p=0.913)	-0.613 (p=0.079)
HF	-0.100 (p=0.798)	0.034 (p=0.931)	0.338 (p=0.374)	-0.371 (p=0.326)	0.298 (p=0.436)	0.546 (p=0.128)
LF/HF	-0.383 (p=0.309)	0.322 (p=0.398)	0.354 (p=0.350)	0.236 (p=0.541)	0.358 (p=0.344)	0.092 (p=0.814)
SD2/SD1	-0.067 (p=0.864)	-0.305 (p=0.425)	-0.692 (p=0.039)*	0.051 (p=0.896)	-0.494 (p=0.176)	-0.639 (p=0.064)

Values represent Spearman’s rank correlation coefficients (r) between post-flight HRV indices and NASA-TLX dimensions after operational maintenance flights, with exact p-values shown in parentheses. HRV, heart rate variability; NASA-TLX, NASA Task Load Index; RR, interval between consecutive R waves; SDNN, standard deviation of normal RR intervals; HRmean, mean heart rate; HRmin, minimum heart rate; HRmax, maximum heart rate; RMSSD, root mean square of successive differences between adjacent RR intervals; LF, low-frequency power; HF, high-frequency power; LF/HF, ratio between low- and high-frequency components; SD2/SD1, ratio between the longitudinal and transverse axes of the Poincaré plot. *p < 0.05 (nominal, uncorrected for multiple comparisons).

Although several correlations reached moderate-to-large magnitudes according to Cohen’s (1988) criteria, only two associations reached nominal statistical significance given the small sample size (n = 9): SD2/SD1 with temporal demand (r = -0.692; p = 0.039) and HRmin with temporal demand (r = -0.667; p = 0.050, borderline). RMSSD also showed a moderate positive correlation with temporal demand (r = 0.650; p = 0.058), approaching but not reaching significance. These findings suggest that, within the operational maintenance condition, temporal demand was the workload dimension most consistently associated with autonomic parameters, although this pattern must be interpreted with caution given the exploratory nature of the analysis.

The remaining associations involving effort and frustration, although reaching moderate magnitudes (e.g., HRmax × effort, r = 0.426; LF × frustration, r = -0.613; HF × frustration, r = 0.546; RMSSD × frustration, r = 0.639; SD2/SD1 × frustration, r = -0.639), did not reach statistical significance (all p > 0.05) and should be interpreted as exploratory trends rather than robust associations.

The remaining associations, including those involving SDNN, LF, and the LF/HF ratio, were of low-to-moderate magnitude and did not reach statistical significance (all p > 0.05).

Overall, these results indicate that, despite the global stability of HRV indices observed between operational conditions, temporal demand showed the most consistent nominal associations with cardiac autonomic parameters during operational maintenance flights. However, only two of the sixty correlations examined across both tables reached statistical significance, and none would survive correction for multiple comparisons. Consequently, these correlations should be interpreted as exploratory and hypothesis-generating rather than confirmatory.

The correlations between HRV indices and perceived workload dimensions after aerial demonstration (AD) flights are presented in **Table 5**. Overall, low to high magnitude associations were observed between autonomic parameters and NASA-TLX components, with particular emphasis on correlations involving spectral HRV components.

**Table 5:** Correlations between HRV indices and NASA-TLX dimensions after aerial demonstration flights.

Variable	Mental demand	Physical demand	Temporal demand	Performance	Effort	Frustration
SDNN	0.102 (p=0.794)	-0.025 (p=0.949)	0.191 (p=0.623)	0.194 (p=0.617)	-0.025 (p=0.949)	0.322 (p=0.398)
HRmean	0.009 (p=0.982)	-0.346 (p=0.362)	-0.131 (p=0.737)	-0.051 (p=0.896)	-0.403 (p=0.282)	0.191 (p=0.623)
HRmin	-0.085 (p=0.828)	-0.219 (p=0.571)	-0.209 (p=0.589)	-0.177 (p=0.649)	-0.420 (p=0.260)	0.139 (p=0.721)
HRmax	0.485 (p=0.186)	0.169 (p=0.664)	0.383 (p=0.309)	0.160 (p=0.681)	0.025 (p=0.949)	0.313 (p=0.412)
RMSSD	0.128 (p=0.743)	0.186 (p=0.632)	0.226 (p=0.559)	0.203 (p=0.600)	0.101 (p=0.796)	0.244 (p=0.527)
LF	0.231 (p=0.550)	-0.140 (p=0.719)	0.420 (p=0.260)	0.420 (p=0.260)	-0.127 (p=0.745)	0.616 (p=0.077)
HF	0.398 (p=0.289)	0.292 (p=0.446)	0.691 (p=0.039)*	0.773 (p=0.015)*	0.521 (p=0.150)	0.496 (p=0.174)
LF/HF	-0.153 (p=0.694)	-0.405 (p=0.280)	-0.209 (p=0.589)	-0.329 (p=0.387)	-0.269 (p=0.484)	-0.322 (p=0.398)
SD2/SD1	-0.068 (p=0.862)	-0.135 (p=0.729)	-0.174 (p=0.654)	-0.329 (p=0.387)	-0.168 (p=0.666)	-0.392 (p=0.297)

Values represent Spearman’s rank correlation coefficients (r) between post-flight HRV indices and NASA-TLX dimensions after aerial demonstration flights, with exact p-values shown in parentheses. HRV, heart rate variability; NASA-TLX, NASA Task Load Index; RR, interval between consecutive R waves; SDNN, standard deviation of normal RR intervals; HRmean, mean heart rate; HRmin, minimum heart rate; HRmax, maximum heart rate; RMSSD, root mean square of successive differences between adjacent RR intervals; LF, low-frequency power; HF, high-frequency power; LF/HF, ratio between low- and high-frequency components; SD2/SD1, ratio between the longitudinal and transverse axes of the Poincaré plot. *p < 0.05 (nominal, uncorrected for multiple comparisons).

Within this exploratory analysis, the largest associations were observed for the HF component, particularly with performance (r = 0.773; p = 0.015) and temporal demand (r = 0.691; p = 0.039), both of which reached nominal statistical significance in this sample. The association between HF and effort (r = 0.521; p = 0.150) did not reach significance despite its moderate magnitude. These findings indicate that, within this single exploratory sample, the high-frequency, predominantly vagal-related component showed the largest associations with perceived workload dimensions during aerial demonstration flights, particularly regarding temporal demand and performance; whether this pattern is stable or replicable across samples cannot be determined from the present data.

The LF component showed moderate positive correlations with temporal demand (r = 0.420), performance (r = 0.420), and frustration (r = 0.616), none of which reached statistical significance (all p > 0.05).

HRmax showed moderate positive correlations with mental demand (r = 0.485) and temporal demand (r = 0.383), neither of which reached statistical significance. HRmin showed a moderate negative correlation with effort (r = -0.420), also not statistically significant; the remaining associations were weak and non-significant.

The LF/HF ratio showed negative correlations with all perceived workload dimensions, and the SD2/SD1 ratio showed a similar pattern of low-magnitude negative correlations; none of these associations reached statistical significance.

Global HRV indices showed weaker associations with perceived workload. SDNN and RMSSD showed low-magnitude, non-significant correlations with all NASA-TLX dimensions.

Overall, these exploratory findings indicate that, during aerial demonstration flights, the HF spectral component showed the most consistent and statistically significant nominal associations with specific dimensions of perceived workload (temporal demand and performance), whereas other HRV indices showed weaker, non-significant associations. As with the operational maintenance condition, these correlations did not survive correction for multiple comparisons and should be interpreted as exploratory.

## 4 Discussion

The present investigation compared the heart rate variability indices obtained during the recovery period immediately after flight and the workload perceived by pilots of the Brazilian Air Force Air Demonstration Squadron during operational maintenance (OM) and air demonstration (AD) missions. By integrating physiological and perceptual measures in a highly specialized operational population, the study broadens the understanding of autonomic recovery processes associated with different aeronautical requirements.

The results showed that aerial demonstration flights were perceived as more demanding, particularly in the temporal demand and effort dimensions assessed by the NASA-TLX (**Figure 2**). In addition, percentage increases were observed in almost all workload dimensions, with the exception of frustration (**Table 2**). This pattern is consistent with the literature characterizing complex flight operations as environments marked by high cognitive demand, the need for simultaneous processing of multiple sources of information, maintenance of situational awareness, and decision-making under substantial time pressure (Mohanavelu et al., 2020; Masi et al., 2023; Almeida et al., 2026).

**Figure 2:**
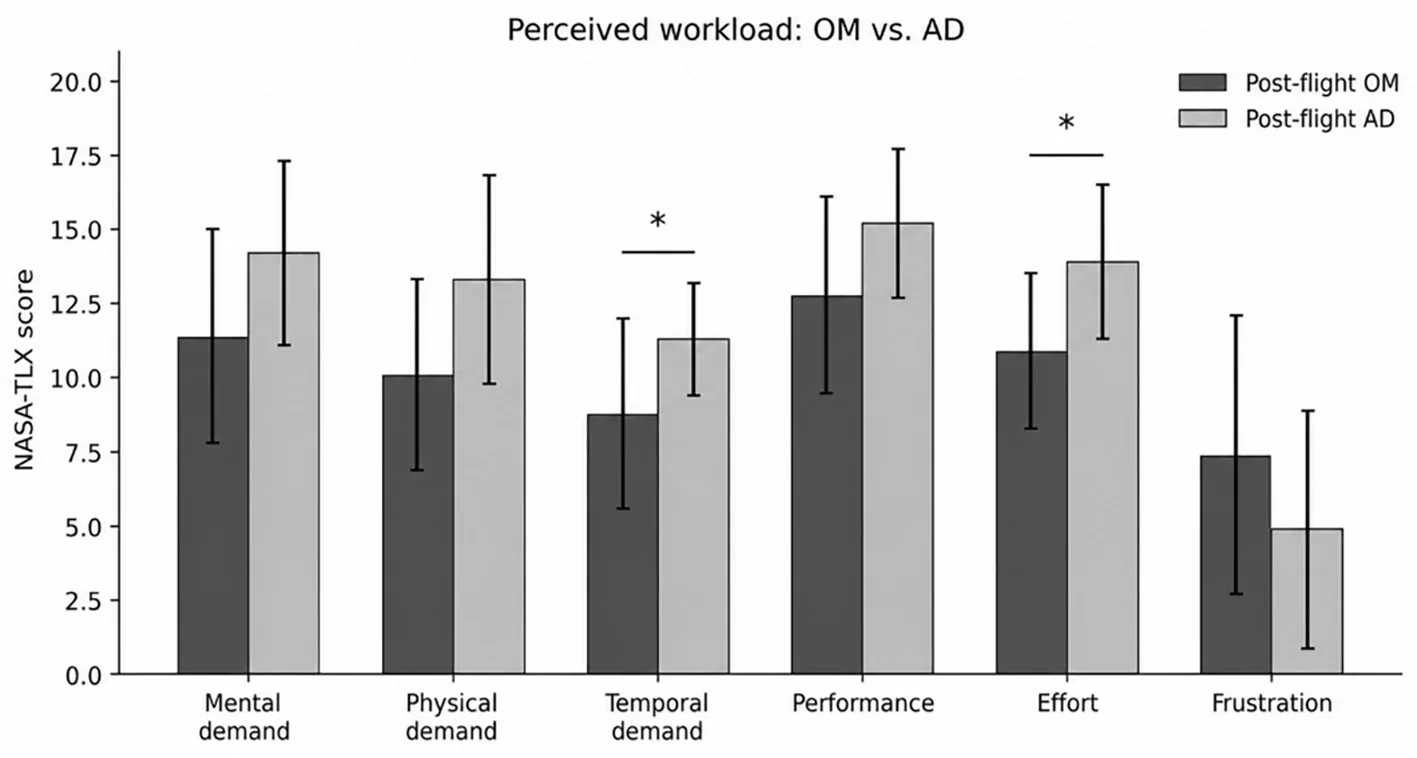
Comparison of perceived workload after operational maintenance and aerial demonstration flights. Image presents the mean workload scores after operational maintenance (OM) and aerial demonstration (AD) flights, with error bars representing the standard deviation. Statistically significant differences were observed for the temporal demand and effort dimensions (p < 0.05), as indicated by asterisks. The remaining dimensions did not differ significantly between the assessed conditions.

Similar findings have been reported in different aviation contexts. Alaimo et al. (2020) observed increased NASA-TLX scores under conditions of greater operational demand, whereas Mohanavelu et al. (2020) demonstrated that the introduction of additional tasks in fighter aircraft simulators significantly increased perceived workload, even in the absence of performance impairment. In Brazilian Air Force military transport pilots, Mendes et al. (2021) also identified a predominance of cognitive over physical demands during operational missions, reinforcing the relevance of mental components in the overall perception of workload.

Despite the increase in perceived subjective load, the autonomic indicators assessed during recovery immediately after landing remained largely preserved across the conditions investigated. RR intervals, SDNN, RMSSD, LF, HF, LF/HF, and SD2/SD1 did not differ significantly between operational maintenance and aerial demonstration flights (**Table 3**). The only significant change was observed in maximum heart rate, which showed an approximate 9% reduction after aerial demonstration flights.

The dissociation observed between perceived workload and autonomic responses during the post-flight recovery period is supported. Koskelo et al. (2024) demonstrated that increased cognitive workload during simulated flights produces specific autonomic adaptations, the magnitude of which depends on the HRV index analyzed. Similarly, the systematic review conducted by Wang et al. (2024) highlighted that changes in mental workload are often not accompanied by consistent changes in traditional HRV indicators, suggesting that different autonomic components may respond differently to operational demands.

The interpretation of these results requires, however, an important consideration of the temporal context of the autonomic evaluation. Although electrocardiographic recording was performed continuously throughout the flight period, HRV analyses were deliberately restricted to the five minutes immediately after landing, allowing direct temporal correspondence with the NASA-TLX application. Thus, the results predominantly reflect the post-flight autonomic recovery and not the physiological response during the execution of the missions. This approach is compatible with the theoretical model of the Vagal Tank proposed by Laborde et al. (2018), according to which autonomic functioning should be interpreted considering three complementary phases: rest, reactivity, and recovery. Since only the recovery phase was analyzed in the present study, the findings should be interpreted as representative of autonomic restoration after different operational demands, and not as a complete characterization of the cardiovascular response during flight.

A complementary analysis extending beyond the recovery window—encompassing pre-flight rest and in-flight reactivity phases—corroborated this interpretation, showing no significant differences between conditions at any of the three phases assessed. This finding adds robustness to the conclusion that the elevated perceived workload associated with aerial demonstration flights was not accompanied by a correspondingly distinct autonomic signature across the mission’s physiological trajectory, at least as captured by the HRV indices examined.

A possible explanation for the absence of significant differences between the assessed conditions in post-flight autonomic indices may be related to the high operational experience of the participants. However, the present design does not allow determination of whether this characteristic was responsible for the observed responses. Accumulated evidence indicates that prolonged exposure to the aviation environment is associated with more efficient cardiovascular responses and more stable autonomic recovery processes after situations of high psychophysiological demand (Newman and Callister, 2008; Sukhoterin et al., 2017). More recent studies support this interpretation. Santos et al. (2022) observed that more experienced military pilots showed better operational performance and more efficient autonomic responses during simulated emergencies, whereas Nascimento et al. (2024) identified differences in cardiovascular responses after real aerobatic flights among pilots with different levels of experience.

The reduction observed in maximum heart rate should be interpreted with caution. Although previous studies suggest that experienced pilots may present differentiated cardiovascular responses during complex operational tasks, the present design does not allow the mechanisms underlying this behavior to be established. Fuentes-García et al. (2021) reported psychophysiological differences related to the operational context of flight, whereas Santos et al. (2022) demonstrated that expertise is associated with greater autonomic efficiency during high-demand situations. Additionally, Wang et al. (2024) highlighted that heart rate often shows greater sensitivity for detecting changes related to mental workload than several traditional HRV indices.

Another relevant aspect refers to the exploratory correlations observed between autonomic indicators and workload dimensions (**Tables 4**, **5**). It should be emphasized that the overwhelming majority of these correlations — including all associations involving RMSSD and LF in both flight conditions, and all associations involving effort in either condition — did not reach statistical significance. Only a small number of nominal associations emerged: SD2/SD1 and HRmin with temporal demand after operational maintenance flights, and HF with temporal demand and performance after aerial demonstration flights. This sparse and largely non-significant pattern is consistent with, and reinforces, the central finding of autonomic stability across conditions; it does not indicate a robust or consistent association between subjective workload and cardiac autonomic modulation, and should not be interpreted as conflicting with the dissociation observed between perceived workload and HRV.

These results reinforce the notion that the relationship between subjective workload and autonomic regulation is multifactorial and not necessarily linear. Alaimo et al. (2020) observed associations between HRV measures and specific NASA-TLX dimensions and also proposed a nonlinear relationship between objective and subjective workload indicators. Taken together, these findings suggest that specific components of the subjective experience may exert a more pronounced influence on certain autonomic mechanisms than global perceived workload.

It should be noted that the present findings diverge, rather than converge, from studies such as Mohanavelu et al. (2020); Koskelo et al. (2024), and Zhu et al. (2023), which reported significant differences in HRV indices between workload conditions. In the present study, no HRV index differed significantly between OM and AD flights in the primary recovery-phase analysis (**Table 3**), and spectral components showed only sparse, largely non-significant correlations with specific NASA-TLX dimensions. This null result may reflect genuine differences between the present operational context — highly experienced pilots assessed during post-flight recovery rather than during task execution — and the simulator- or in-task-based paradigms used in most previous studies, and should not be interpreted as evidence of shared sensitivity of spectral HRV components across studies. Nonetheless, the practical relevance of spectral analysis lies in the fact that these components may still provide complementary information on the organization of the autonomic response in complex operational contexts, warranting further investigation, ideally including in-flight (reactivity-phase) measurement, before conclusions about their sensitivity as workload markers can be drawn. Additionally, multimodal approaches combining HRV with other physiological markers have shown greater capacity to explain variations in perceived workload than the isolated use of a single physiological measure (Durantin et al., 2014; Boffet et al., 2025).

The reduction in frustration observed during aerial demonstration flights, despite the simultaneous increase in the other operational demands, is a particularly interesting finding. This result suggests that highly complex tasks may not necessarily be interpreted as negative experiences by highly trained individuals. In contexts of high expertise, operational challenges may be perceived as inherent elements of professional performance, thereby reducing the perception of discomfort or inability even in the presence of greater cognitive and motor demands.

Overall, the results suggest, on an exploratory basis, that the increase in perceived workload during aerial demonstration flights was not accompanied by substantial changes in most of the autonomic indicators assessed. The combination of higher subjective operational demand, reduced maximum heart rate, and maintained HRV indices suggests that these pilots possess a high capacity for physiological adaptation to the demands of the aeronautical environment. These findings contribute to advancing knowledge on human factors in military aviation and reinforce the importance of integrating subjective and physiological measures for monitoring operational performance in populations exposed to highly complex environments.

## 5 Study limitations

Some limitations should be considered when interpreting the results. First, the small sample size (n = 9) limits the statistical power of the analyses and increases susceptibility to Type I and Type II errors. However, this sample represents approximately 60% of the active operational personnel of the Brazilian Air Force Aerial Demonstration Squadron, constituting a highly specialized, naturally restricted population that is difficult to access for scientific investigation.

Another important limitation refers to the timing of HRV assessment. Autonomic parameters were obtained during the five minutes immediately after landing, predominantly reflecting cardiovascular recovery responses to flight rather than autonomic adaptations occurring during the execution of aerial maneuvers. Therefore, the results should be interpreted as indicators of the post-flight autonomic state and do not allow direct inferences regarding the physiological dynamics developed throughout the mission.

Additionally, the adopted design did not allow control of factors that may modulate autonomic activity, such as sleep quality, nutritional status, accumulated operational load, previous stress level, and circadian variation. Although assessments were conducted according to standardized procedures, these variables may influence HRV indices and should be considered in future studies. The study also did not include a standardized baseline resting condition for comparison of autonomic responses observed after flights. Consequently, the results allow us to compare the recovery profiles between the two types of mission evaluated, but not to quantify the absolute degree of physiological deviation in relation to resting conditions or daily activities. Future studies should incorporate baseline pre-flight measurements to more comprehensively characterize the autonomic responses associated with the different phases of aerial activity.

Another limitation refers to the absence of counterbalancing of the experimental sequence. All participants initially carried out operational maintenance flights and later air demonstration flights. Although a minimum interval of seven days was adopted between evaluations, it is not possible to completely exclude potential effects of psychological order, familiarization or anticipation. This strategy was necessary to preserve the operational routine of the Air Demonstration Squadron and ensure the logistical feasibility of data collection in a real environment.

Finally, the specificity of the sample should be emphasized. Participants belonged to an elite military aviation unit characterized by a high level of training and operational experience. Although this characteristic increases the ecological validity of the findings, it limits their generalizability to other populations of military pilots, civilian aviators, or individuals with less flight experience.

Future investigations involving multicenter samples, continuous physiological monitoring during flight, and longitudinal designs may expand the understanding of autonomic and perceptual responses associated with different operational demands in military aviation.

## 6 Conclusion

Aerial demonstration flights were associated with higher levels of perceived workload, particularly in the temporal demand and effort dimensions, when compared with operational maintenance flights. Despite this increase in perceived operational demands, no significant differences were observed in most HRV indices assessed during immediate post-flight recovery, with only a reduction in maximum heart rate being identified after aerial demonstration flights.

These results suggest that pilots from the Aerial Demonstration Squadron may exhibit relative autonomic stability during the post-flight recovery period, even when exposed to greater subjective workload during missions of higher operational complexity.

From an applied perspective, the findings reinforce the importance of integrating physiological and perceptual measures for monitoring human factors in real aviation environments. In addition, they provide evidence obtained from a rare and highly specialized operational population, contributing to the advancement of knowledge on psychophysiological monitoring, human performance, and operational safety in military aviation. 

## Data Availability

The raw data supporting the conclusions of this article will be made available by the authors, without undue reservation.
